# The tail domain of PRRSV NSP2 plays a key role in aggrephagy by interacting with 14-3-3ε

**DOI:** 10.1186/s13567-020-00816-7

**Published:** 2020-08-18

**Authors:** Shengliang Cao, Jiaqi Liu, Guofei Ding, Qingyuan Shao, Bin Wang, Yingchao Li, Jian Feng, Yuzhong Zhao, Sidang Liu, Yihong Xiao

**Affiliations:** 1 Department of Fundamental Veterinary Medicine, College of Animal Science and Veterinary Medicine, Shandong Agricultural University, 61 Daizong Street, Tai’an, 271018 Shandong China; 2 Shandong Provincial Key Laboratory of Animal Biotechnology and Disease Control and Prevention, Shandong Agricultural University, Tai’an, Shandong China; 3 Shandong Provincial Engineering Technology Research Center of Animal Disease Control and Prevention, Shandong Agricultural University, Tai’an, China

**Keywords:** PRRSV, NSP2, Aggrephagy, 14-3-3ε, Tail domain of NSP2

## Abstract

Porcine reproductive and respiratory syndrome (PRRS) caused by PRRS virus (PRRSV) is one of the most severe swine diseases that affects almost all swine-breeding countries. Nonstructural protein 2 (NSP2) is one of the most important viral proteins in the PRRSV life cycle. Our previous study showed that PRRSV NSP2 could induce the formation of aggresomes. In this study we explored the effects of aggresome formation on cells and found that NSP2 could induce autophagy, which depended on aggresome formation to activate aggrephagy. The transmembrane and tail domains of NSP2 contributed to aggrephagy and the cellular protein 14-3-3ε played an important role in NSP2-induced autophagy by binding the tail domain of NSP2. These findings provide information on the function of the C-terminal domain of NSP2, which will help uncover the function of NSP2 during PRRSV infection.

## Introduction

Porcine reproductive and respiratory syndrome (PRRS) caused by PRRS virus (PRRSV) is one of the most severe swine diseases and is responsible for substantial economic losses [1]. According to the OIE database (https://www.oie.int/wahis_2/public/wahid.php/Diseaseinformation/Diseasetimelines), 64 of 210 countries or areas have reported PRRSV infections, including China, European Union, the United States of America, Canada, and Russia, which were among the top 10 pork-producing countries. It is estimated that the economic impact of PRRSV on pork producers in the United States alone is more than $560 million annually [2].

PRRSV is an enveloped single-stranded positive sense RNA virus belonging to the family *Arteriviridae* and order *Nidovirales*. The classification of PRRSV is updated recently and divided into two species to accommodate the clear divergence of the European type (PRRSV-1) and American type (PRRSV-2) [3]. The PRRSV genome is ~ 15 Kb with 11 open reading frames (ORFs) encoding 14 nonstructural proteins and eight structural proteins [4–7]. Two polyproteins, PP1a and PP1ab, are encoded by ORF1a and ORF1b, respectively, and are processed into at least 14 nonstructural proteins (NSPs) by four ORF1a-encoded proteinases called NSP1α, NSP1β, NSP2, and NSP4 [8–11]. NSP2 is the largest product, and contains a cysteine protease (PL2) domain, a central hypervariable (HV) domain, and a transmembrane (TM) domain followed by a tail domain [11, 12].

The PL2 domain is a member of the ovarian tumor (OTU) protease superfamily and is responsible for the cleavage of PP1a between NSP2 and NSP3 [13, 14]. This domain also exhibits deubiquitinating and interferon-antagonism functions, and inhibits the antiviral function of interferon-stimulated gene 15 to block innate antiviral responses [15–17]. The HV domain is the most variable domain in the viral genome. Mutations, insertions, and deletions occur in this domain, which affects the overall length of the genome [18–20].

The highly pathogenic and NADC30-like PRRSV-2 emerged in China in 2006 and 2013 and contained deletions of 30 and 131 amino acids in the NSP2 gene, respectively [21–23]. Most concerning was the emergence of a recombinant PRRSV-2 between the HP-PRRSV and NADC30-like strains in the HV domain of NSP2 [24–26]. It was recently reported that the HV domain is associated with viral cellular tropism to primary porcine alveolar macrophages and contributes to targets of neutralizing antibodies [27, 28]. Little information is available on the TM and tail domains.

In eukaryotic cells, protein aggregates formed by incorrectly folded proteins are delivered to the microtubule organizing center (MTOC) along microtubules via dynein-dependent retrograde transport [29]. When the degradative capacity of proteasomes is exceeded, protein aggregates accumulate in perinuclear inclusions called aggresomes [30, 31]. Viral infections always produce large amounts of viral proteins that cannot be folded and form an aggresome. Aggresomes are thought to provide a physical scaffold to concentrate viral components and thereby increase the efficiency of replication [32]. Viruses use different strategies to make full use of aggresomes during their life cycle. For example, influenza A virus uses the aggresome-processing machinery for host-cell entry [33], while Asfarvirus, iridoviruses, poxviruses, retroviruses, and herpesviruses use aggresomes as virus assembly sites to facilitate replication and assembly [32, 34, 35]. Virus-infected cells always evoke autophagy to degrade the aggresomes for self-protection. This process is called aggrephagy [31, 36, 37].

Our previous study showed that PRRSV-2 NSP2 could induce the formation of aggresomes [38, 39], a process in which cellular protein 14-3-3 played a role. It has been confirmed that PRRSV-2 infections can induce autophagy to facilitate infection [40–43]. These findings raised the questions of whether autophagy caused by PRRSV-2 is induced by the NSP2 aggresome and what the role of 14-3-3 is during this process.

## Materials and methods

### Cell culture and viruses

293T cells (human embryo kidney cells), Marc-145 cells (PRRSV-susceptible cell line derived from African monkey kidney), BHK21 cells (baby hamster Syrian kidney), and Vero E6 cells (African green monkey kidney cells) were obtained from the China Center for Type Culture Collection (Wuhan, China). Cells were cultured in Dulbecco’s modified Eagle’s medium (DMEM; Gibco Invitrogen, Carlsbad, CA, USA) supplemented with 10% (v/v) fetal bovine serum (FBS; Biological Industries, Beit HaEmek, Israel) and 1% (v/v) penicillin–streptomycin (Solarbio Life Science, Beijing, China). All cells were grown at 37 °C in a humidified incubator with 5% CO_2_. A high-pathogenic PRRSV-2 (HP-PRRSV) strain (strain TA-12; GenBank No. HQ416720) was preserved by our team. A low-pathogenic PRRSV-2 (LP-PRRSV) strain, CH-1R, was kindly provided by Professor Enmin Zhou (Northwest A&F University, China).

### Plasmids and transfections

The full-length and truncated NSP2 genes were cloned into pEGFP-C1 vector which was tagged by green fluorescent protein (GFP), and GFP-NSP2, OTU-GFP (aa 47–159), HV-GFP (aa 160–844), TM-GFP (aa 845–1018), and Tail-GFP (aa 1019–1194) were generated as previously described [38]. FLAG-NSP2 was generated using the pCDNA3.0-FLAG vector by inserting NSP2 between the BamH I and Not I restriction sites. The NSP3 gene of TA-12 was cloned into pEGFP-C1 using Bgl II and Hind III restriction sites to generate GFP-NSP3. The PDsRed2-C1-LC3 plasmid was kindly provided by Jiuqiang Wang (Institute of Zoology, Chinese Academy of Sciences). The mutant plasmids for NSP2-Tail were generated via PCR and harbored mutations encoding the substitutions R991K and A994E (numbered according to the NSP2 sequence of HP-PRRSV strain TA-12), and named KTAP and RTEP, respectively. NSP2-Tail without mutations was referred to as the wild type (WT).

Transfections were performed using X-tremeGENE HP DNA transfection reagent (Roche Applied Science, Basel, Switzerland) according to the manufacturer’s instructions. Then new DMEM complete medium containing 10% FBS was replaced before transfection. Cells were collected for Western blot analyses at 12–36 h after transfection.

### Antibodies and reagents

Antibodies and their manufacturers were as follows: anti-β-actin (AP0060; Bioworld Technology, St. Louis Park, MN, USA), anti-α glyceraldehyde 3-phosphate dehydrogenase (GAPDH) (1:5000) (AT0002; CMCTAG, Dover, DE, USA), anti-GFP (1:5000) (AT0028; CMCTAG), anti-FLAG (1:5000) (AT0022; CMCTAG), anti-LC3 (1:3000) (L7543; Sigma-Aldrich), anti-p62 (1:5000) (P0067; Sigma-Aldrich), and anti-14-3-3ε (1:800) (sc-23957; Santa Cruz Biotechnology, Santa Cruz, CA, USA). Anti-NSP2 antibodies were prepared by immunizing New Zealand white rabbits with a recombinant protein composed of the N-terminal 180 amino acids of NSP2 (NSP2-180). The monoclonal antibody against PRRSV-2 nucleocapsid (N) protein 6D10 was prepared in our laboratory [44]. Horseradish-peroxidase-conjugated anti-mouse or anti-rabbit secondary antibodies were purchased from Jackson Laboratories (1:5000) (West Grove, PA, USA).

Complete™ protease inhibitor cocktail (04693132001) was purchased from Roche (Basel, Switzerland), the aggresome detection kit (ab139486) was purchased from Abcam (Cambridge, UK), and nocodazole (M1404) and MG132 (C2211) were obtained from Sigma-Aldrich.

### Confocal fluorescence microscopy

Marc-145 cells, 293T cells, and Vero E6 cells were seeded onto coverslips in 24-well plates and transfected with plasmids using X-tremeGENE HP DNA transfection reagent (Roche, Basel, Switzerland). Marc-145 cells were either mock infected or infected with TA-12 or CH-1R at a multiplicity of infection of 0.01. Cells were harvested at the indicated time points and fixed with 4% paraformaldehyde (Beyotime Biotech, Shanghai, China) for 15 min. They were then permeabilized with 0.1% Triton X-100 (Solarbio Life Science) in phosphate-buffered saline (PBS) for 15 min, and blocked with 1% bovine serum albumin (BSA; Solarbio Life Science) in PBS for 1 h. After washing three times with PBS, cells were incubated with primary antibodies or aggresome detection reagent diluted in PBS for 2 h. Cells were then washed and incubated with fluorescently-labeled secondary antibodies conjugated to fluorescein isothiocyanate or Cy3. Cell nuclei were stained with 4′,6-diamidino-2-phenylindole (DAPI; Invitrogen). All laser scanning images were obtained using a fluorescence microscope (Leica, SPE, Buffalo Grove, IL, USA).

### Transmission electron microscopy

293T cells were transfected with GFP-NSP2 and pEGFP-C1 plasmids. Following transfection for 24 h, cells were harvested and centrifuged in clean 2-mL round-bottomed centrifuge tubes. After washing with ice-cold PBS twice, cells were pelleted and fixed in fresh ice-cold 3% glutaraldehyde. Samples were rinsed, fixed with 1% osmium tetroxide (OsO_4_), rinsed again, dehydrated, impregnated, and embedded in Epon812 resin (Electron Microscopy Sciences, Hatfield, PA, USA) according to conventional transmission electron microscopy (TEM) sample preparation procedures. The processed samples were then sectioned using an LKB-V ultra-thin microtome and stained with uranyl acetate and lead citrate. Sections were observed using a transmission electron microscope (JEOL-1200EX; JEOL, Tokyo, Japan) and recorded using an electron microscope (MORADA-G2; EMSIS GmbH, Münster, Germany).

### GFP pull-down assays and Western blot analysis

Cells in 10-cm dishes were transfected with either empty vector, WT NSP2-Tail-GFP (WT), or EGFP-tagged NSP2-Tail mutant plasmid (RTEP or KTAP), with four dishes per plasmid. Cells harvested at 24 h after transfection were extracted in ice-cold lysis buffer (10 mM Tris/HCl, pH 7.5, 150 mM NaCl, 0.5 mM EDTA, 0.5% NP-40) supplemented with Complete™ protease inhibitor cocktail (Roche) for 30 min on crushed ice and then centrifuged at 20,000 × *g* for 20 min at 4 °C. For GFP pull-down assays, whole-cell extracts were incubated with equilibrated GFP-trap beads for 5 h at 4 °C (gta-20; Beijing Lab, Beijing, China). After centrifugation at 2500 × *g* for 2 min at 4 °C, the beads were washed three times with dilution buffer (10 mM Tris/HCl, pH 7.5, 150 mM NaCl, 0.5 mM EDTA) supplemented with Complete™ protease inhibitor cocktail. Precipitated proteins were eluted with 100 µL of 5 × sodium dodecyl sulfate (SDS) loading buffer by boiling at 95 °C for 6 min.

Marc-145, 293T, and BHK21 cells were lysed in RIPA buffer (P0013B; Beyotime, Shanghai, China) supplemented with protease inhibitor cocktail (CW2200, CWBIO, Beijing, China). Protein concentrations were measured with a Pierce™ BCA protein assay kit (23227; ThermoFisher, Shanghai, China).

Equal amounts of precipitated protein or cell lysates were resolved using 10% or 12% SDS–polyacrylamide gel electrophoresis and then transferred to a polyvinylidene difluoride (PVDF) membrane (Millipore Corporation, Bedford, MA, USA) using a Bio-Rad Trans-Blot apparatus (Bio-Rad Laboratories, Hercules, CA, USA) and standard procedures. PVDF membranes were blocked with 5% (W/V) BSA in PBST (PBS with 1% Tween-20) for 1 h at room temperature, and probed with the indicated primary antibodies in blocking buffer at 4 °C overnight. Following overnight incubation with primary antibodies, membranes were incubated for 1 h with appropriate horseradish-peroxidase-conjugated anti-mouse or anti-rabbit secondary antibodies. Proteins were visualized using the Clarity™ Western ECL substrate (170-5060; Bio-Rad Laboratories) and detected using a Western blot fluorescence imager (Vilber Fusion FX7; Vilber Lourmat, Collégien, France). The density of the protein bands was measured using Fusion analysis software in the Vilber Fusion FX7 imaging system. Band densities were determined after subtracting the density of the GAPDH or β-actin bands.

### Nocodazole toxicity analysis and treatment

Cytotoxicity was measured using the Cell Counting Kit-8 (CCK-8) assay (TransGen Biotech, Beijing, China) according to the manufacturer’s instructions. Marc-145 cells and 293T cells were seeded at a density of 5 × 10^3^ cells per well in complete medium in 96-well plates. After 12 h of culture, nocodazole was added to each well at specific concentrations and incubated for 48 h at 37 °C. Dimethyl sulfoxide-treated cells were included as controls. After treatment, the medium was removed and changed to 100 µL of PBS containing 10% CCK-8 solution. After incubation for 2 h at 37 °C, cell viability was detected by measuring the absorbance at 450 nm using a microplate reader (Bio-Rad model 680). The above experiment was repeated three times.

After infection with PRRSV-2, Marc-145 cells were incubated in maintenance medium containing 0.08, 0.16, or 0.32 μg/mL nocodazole for 24 h, and then harvested for Western blot analysis. 293T cells were transfected with pEGFP-C1 and GFP-NSP2 plasmids for 6 h. The cells were incubated with different concentrations of nocodazole (0.08, 0.16, and 0.32 μg/mL) for 4 h, and then collected at 24 h after transfection for Western blot analysis. 293T cells were also incubated with 0.32 μg/mL nocodazole for 4 h, and then collected at different time points for Western blot analyses.

### Statistical analysis

Statistical analysis was performed using the SPSS 23.0 software package (SPSS Inc., version 23.0; Chicago, IL, USA). All data are expressed as the mean ± standard deviation (SD) from at least three biological replicates (n) for each condition. Statistical differences between groups were assessed using Student’s *t* test. *P*-values less than 0.05 were considered to indicate a statistically significant difference (^*^P < 0.05, ^**^P < 0.01, and ^***^P < 0.001).

## Results

### NSP2 can induce autophagy

**Figure 1:**
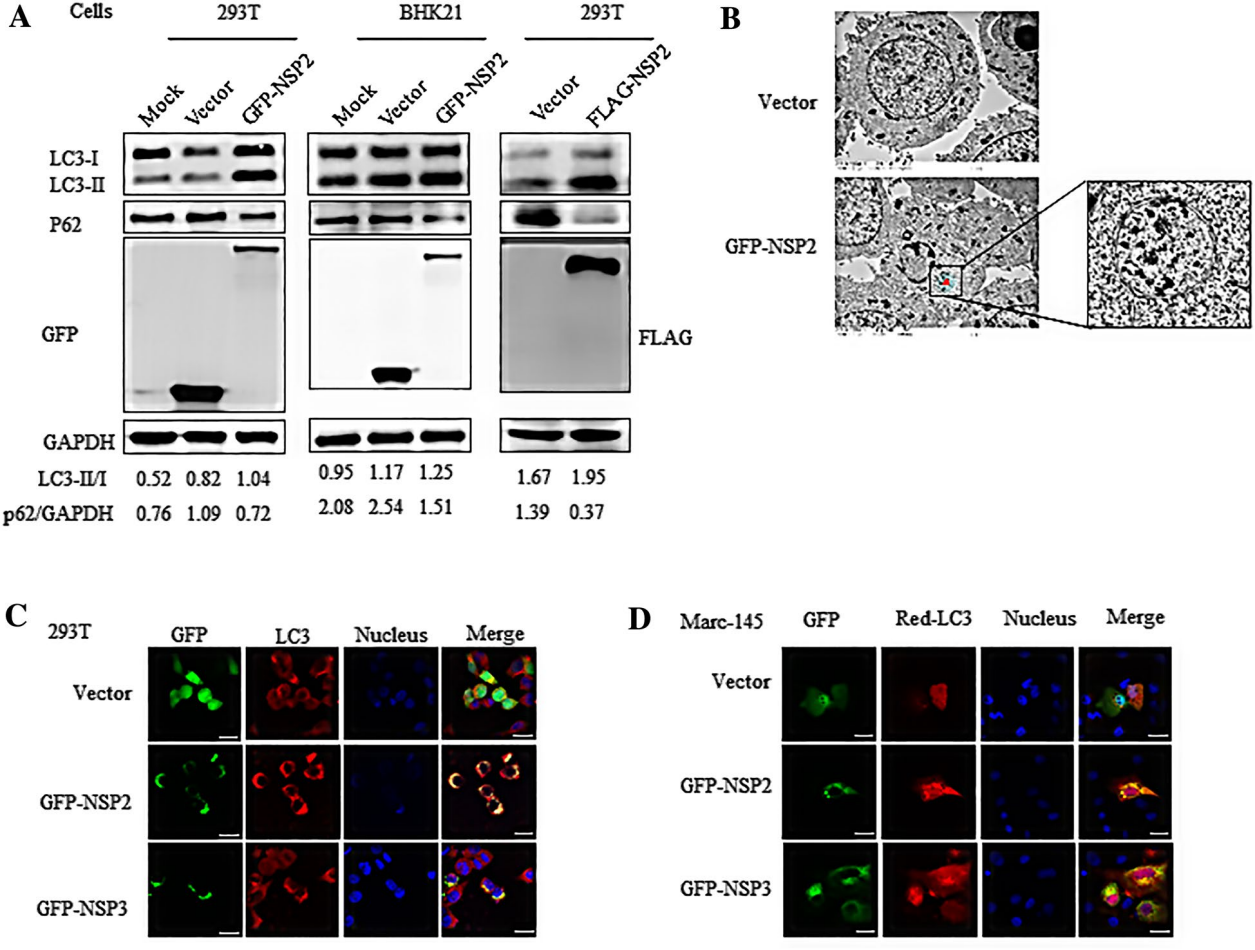
**NSP2 induces autophagy.** **A** Western blot analyses of the effect of NSP2 expression on LC3-II, LC3-I, and p62. 293T and BHK21 cells were transfected with empty vector, GFP-NSP2, or FLAG-NSP2. At 24 h after transfection, cell lysates were subjected to western blotting to analyze autophagy using anti-LC3 and p62 antibodies. The expression of NSP2 was verified by western blotting using anti-GFP or anti-FLAG antibodies. GAPDH served as the protein loading control. **B** Double-membrane phagophores structures were observed using an electron microscope. 293T cells were transfected with empty vector and GFP-NSP2 for 24 h. The cells were then fixed and sectioned, and the sections were observed using a transmission electron microscope. The red arrowheads indicate the double-membrane structure of autophagosomes. Co-localization of NSP2 and LC3 in 293T cells (**C**) and Marc-145 cells (**D**). 293T or Marc-145 cells were transfected with empty vector, GFP-NSP2, and GFP-NSP3 and were analyzed via confocal microscopy using anti-LC3B antibodies. EGFP-tagged proteins are green and LC3 proteins are red. Yellow represents the co-localization between proteins in the merged images. Cells transfected with the GFP vector served as negative control. Bar size: 25 µm.

Autophagy is a defense mechanism for clearance of toxic proteins, including viral proteins. However, autophagy-mediated clearance of aggresome-like inclusions is a selective phenomenon [45]. To determine whether NSP2-induced aggresomes could induce autophagy, we firstly studied whether NSP2 could mediate autophagy. Full-length NSP2 with GFP or FLAG tags was expressed in 239T or BHK21 cells. Western blot results revealed that the LC3-II/LC3-I ratio increased in the NSP2-expressing cells, while p62 levels decreased (Figure 1A). Electron microscopy analyses also revealed a double membrane-bound compartment in 293T cells expressing NSP2 (Figure 1B). NSP2 also co-localized with LC3 (Figure 1C). Taken together, these findings indicate that NSP2 could induce autophagy.

### NSP2-induced autophagy depends on aggresome formation

**Figure 2:**
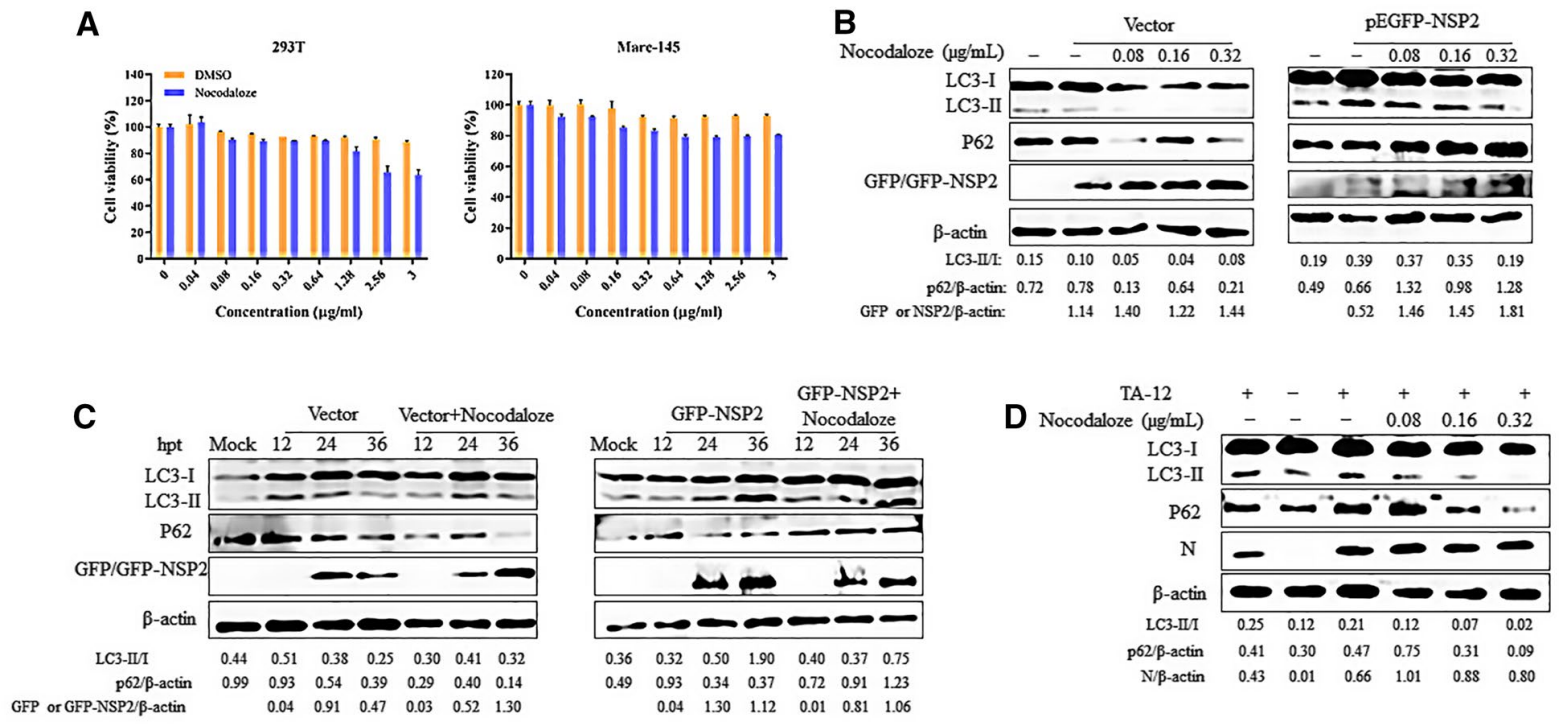
**NSP2-induced autophagy depends on aggresome formation.** **A** The cytotoxicity of nocodazole was determined using the CCK-8 assay. Monolayers of Marc-145 cells and 293T cells in 96-well plates were treated with nocodazole at different concentrations for 48 h, after which the CCK-8 reagent was added to each well. After incubation for 2 h at 37 °C, cell viability was detected by measuring the absorbance at 450 nm. **B** 293T cells were transfected with empty vector and GFP-NSP2. After 6 h, cells were left untreated or treated with nocodazole (0.08, 0.16, or 0.32 µg/mL) for 4 h. At 24 h after transfection, cell lysates were used for western blot analyses with antibodies against β-actin, GFP, p62, and LC3. **C** 293T cells were transfected with empty vector and GFP-NSP2. After 6 h, cells were left untreated or treated with nocodazole (0.32 µg/mL) for 4 h. Cell lysates were harvested at 12 h, 24 h, and 36 h after transfection and analyzed by western blot using antibodies against β-actin, GFP, p62, and LC3. **D** Marc-145 cells were infected with HP-PRRSV (strain TA-12) at a multiplicity of infection of 0.1 for 1 h at 37 °C, after which the medium was replaced with maintenance medium containing 0.08, 0.16, or 0.32 μg/mL nocodazole. Cells were harvested 24 h later and analyzed by western blot with a monoclonal antibody (6D10) targeting the PRRSV N, or anti-β-actin, anti-GFP, anti-p62, and anti-LC3 antibodies.

Aggresomes are the perinuclear inclusion bodies formed around the MTOC by active minus-end-directed transport of misfolded proteins on microtubules [32]. To further confirm the relationship between NSP2-induced autophagy and NSP2-induced aggresomes, nocodazole, which induces microtubule depolymerization, was used as an inhibitor of aggresome formation. The cytotoxicity of nocodazole was confirmed by CCK-8 assays (Figure 2A). 293T cells were transfected with GFP-NSP2 or empty vector and then treated with 0.08, 0.16, or 0.32 µg/mL nocodazole. The results revealed that the LC3-II/LC3-I ratio was reduced while the level of p62 increased in a dose-dependent manner (Figure 2B). 293T cells were transfected with GFP-NSP2 and then treated with 0.32 µg/mL nocodazole. Cells were collected at 12, 24, and 36 h after transfection for western blot analysis. The western blot results also revealed that the LC3-II/LC3-I ratio was decreased, while levels of p62 increased (Figure 2C). These results show that disruption of aggresomes could decrease autophagy. To further confirm this finding, Marc-145 cells were infected with TA-12 and treated with nocodazole. The results showed that the LC3-II/LC3-I ratio and p62 levels were decreased compared to untreated cells (Figure 2D). All these results showed that NSP2-induced autophagy depends on aggresome formation to activate aggrephagy.

### The TM and tail domains of NSP2 contribute to aggresome formation

**Figure 3:**
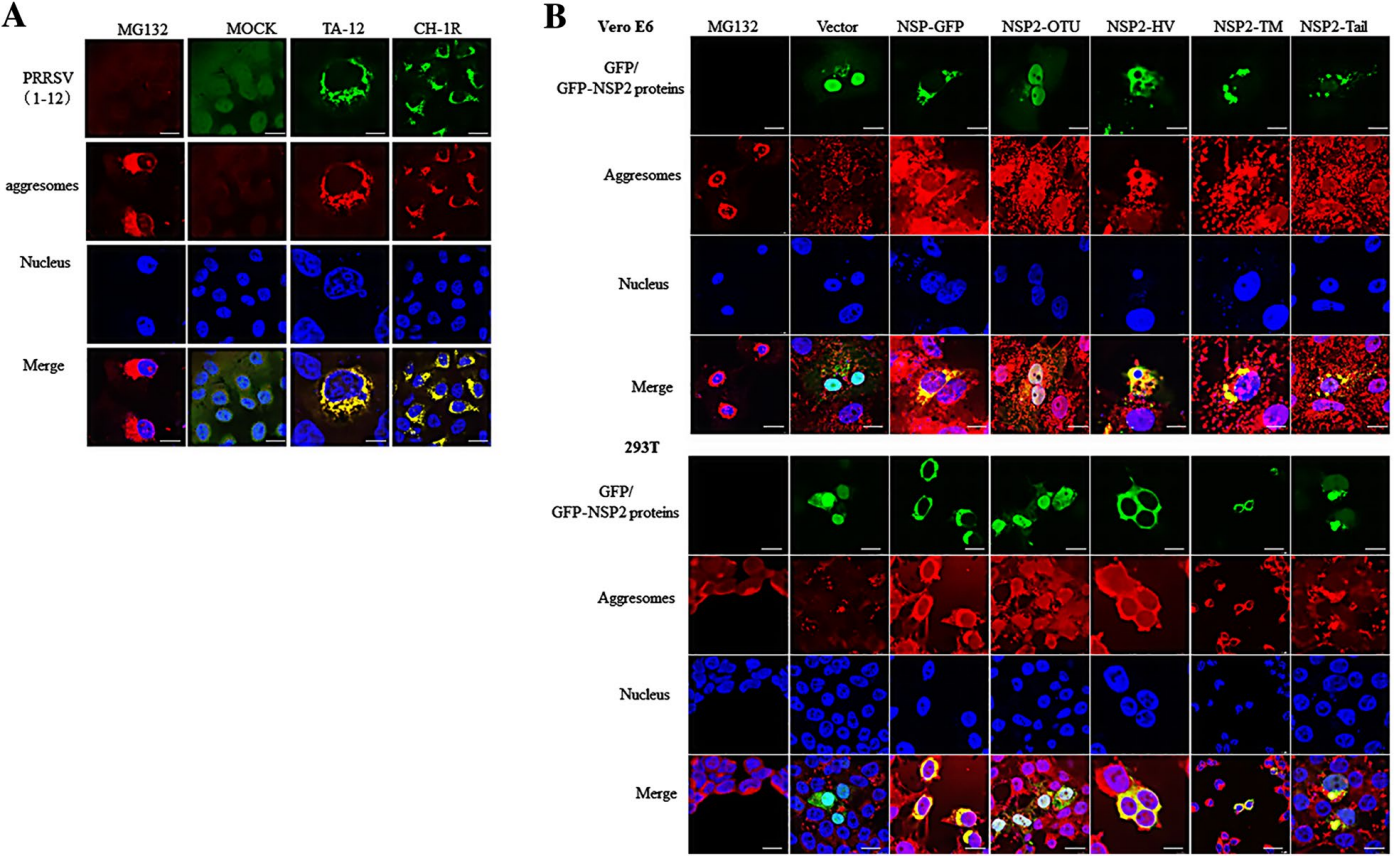
**TM and tail domains of NSP2 co-localize with aggresomes.** **A** Confocal microscopy of Marc-145 cells mock infected or infected for 24 h with TA-12 and CH-1R at a multiplicity of infection of 0.01 (left margin), then stained for PRRSV (green) with anti-NSP2 antibodies and aggresomes (red) with aggresome detection reagent. The nucleus was stained with the DNA-binding dye DAPI (blue). Before harvesting, positive controls were set by incubation with diluted proteasome inhibitor (MG-132, 5 µM) for 18 h under normal cell culture conditions. **B** 293T and Vero E6 cells were transfected with NSP2-GFP, OTU-GFP, HV-GFP, TM-GFP, Tail-GFP, and empty vector (green). At 24 h later, cells were fixed and stained with aggresome detection reagent (red). The treatment method for positive cells is shown in (**A**). Yellow in the merged images represents the formation of aggresomes. Bar size: 25 µm.

Our previous studies revealed that NSP2 can induce the formation of aggresomes [38]. To further confirm the formation of aggresomes, Marc-145 cells were infected with TA-12 or the LP-PRRSV strain. Both strains co-localized with aggresomes (Figure 3A).

NSP2 contains OTU, HV, TM, and tail regions. To test which region(s) contribute to the formation of aggresomes, NSP2 constructs containing the four gene fragments were transfected into Vero E6 and 293T cells. The TM and tail regions had a dotted appearance under confocal microscopy and co-localized with aggresomes in cells (Figure 3B).

### The TM and tail domains of NSP2 contribute to aggrephagy

**Figure 4:**
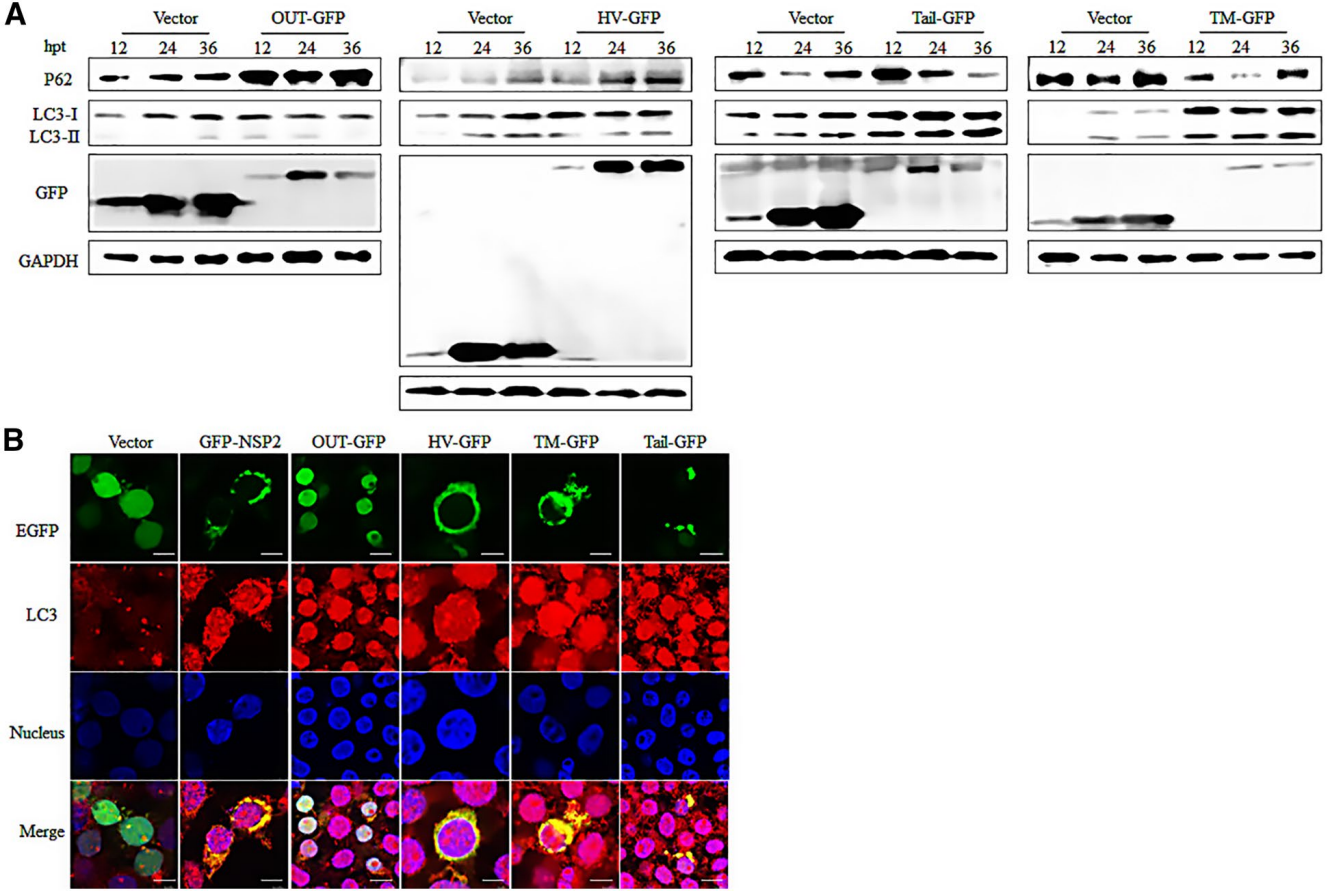
**The TM and tail domains of NSP2 contribute to aggresome autophagy.** **A** 293T cells were transfected with empty vector, GFP-NSP2, and NSP2 truncated plasmids (NSP2-GFP, OTU-GFP, HV-GFP, TM-GFP and Tail-GFP). At 12 h, 24 h, and 36 h after transfection, cell lysates were harvested and used for western blot analyses with the indicated antibodies (anti-GAPDH, anti-GFP, anti-p62, and anti-LC3). **B** 293T cells were transfected with an empty vector, GFP-NSP2, and NSP2 truncated plasmid (NSP2-GFP, OTU-GFP, HV-GFP, TM-GFP, and Tail-GFP). The cells were then analyzed by confocal microscopy. GFP-tagged proteins are shown in green and endogenous LC3 proteins are shown in red. Yellow in the merged images represents the co-localization between proteins. Bar size: 25 µm.

Marc-145 cells were infected with TA-12 and CH-1R strains, respectively. The results revealed that both viruses could co-localize with aggresomes. Thus, to test which region(s) of NSP2 contribute to autophagy, the truncated constructs were transfected into 293T cells and whole-cell lysates were analyzed by western blot. The results show that the tail and TM domains of NSP2 could induce an increase in the LC3-II/LC3-I ratio and a reduction in p62 levels (Figure 4A). The TM and tail domains appeared as dotted particles and co-localized with LC3 (Figure 4B). Taken together, these results indicate that the TM and tail domain of NSP2 contribute to aggrephagy.

### The tail of NSP2 induces autophagy by binding 14-3-3ε

**Figure 5:**
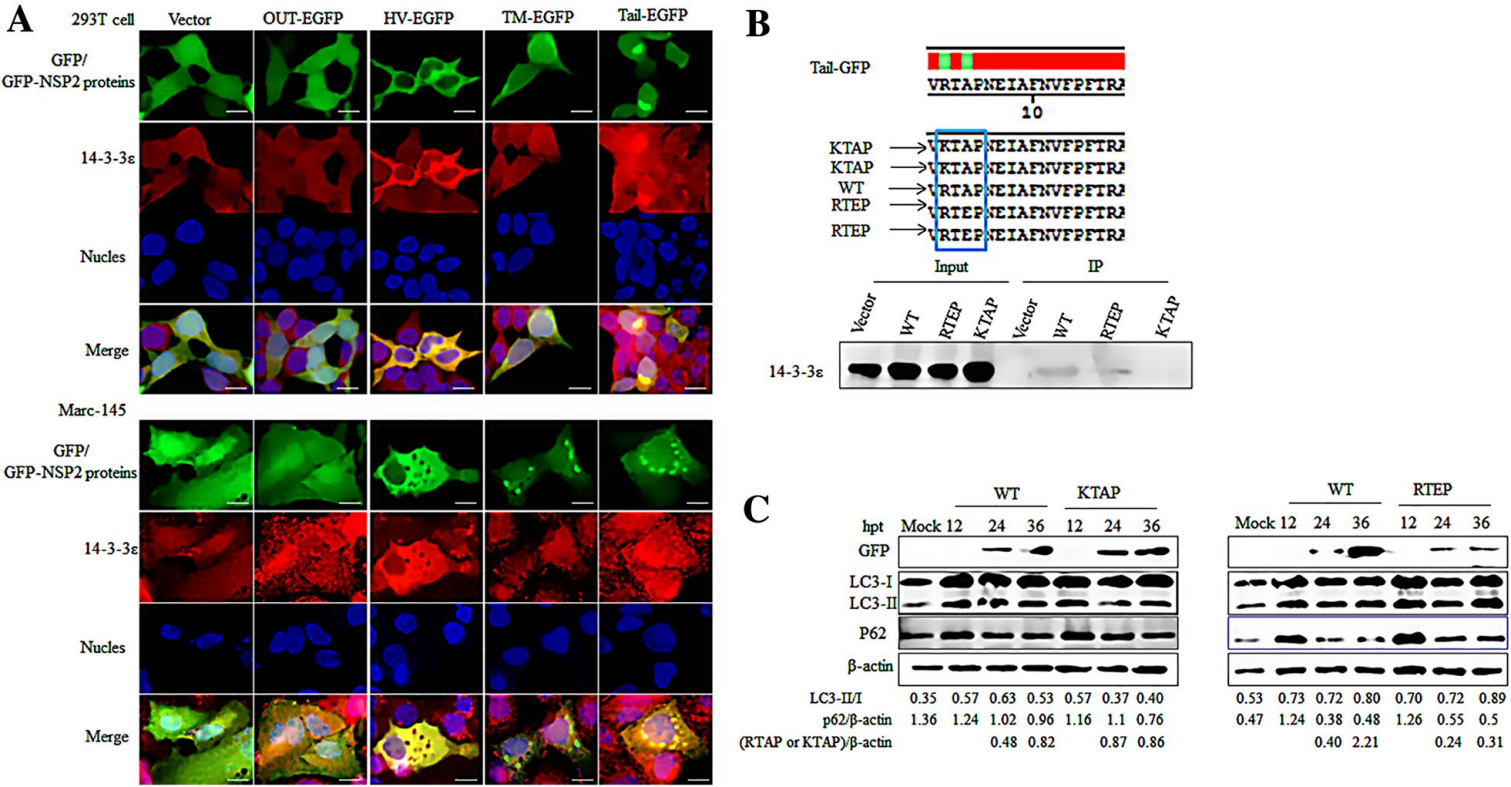
**The tail domain of NSP2 induces autophagy by binding 14-3-3ε.** **A** Immunofluorescence microscopy showing the co-localization of 14-3-3ε and NSP2 protein domains. The co-localization of truncated EGFP-tagged NSP2 plasmids (NSP2-GFP, OTU-GFP, HV-GFP, TM-GFP, and Tail-GFP) with 14-3-3ε was visualized in transfected 293T cells and Marc-145 cells by immunofluorescence microscopy. GFP-tagged proteins are shown in green and endogenous LC3 proteins are shown in red. Yellow in the merged images represents co-localization between proteins. Bar size: 25 µm. **B** The amino acid sequence of the NSP2 tail region containing the 14-3-3ε-binding motif, and the RTEP and KTAP (blue box to mark the location) tail mutants (top). 293T cells were transfected with an empty vector, wild-type Tail-GFP plasmids (WT) or mutant GFP-tagged tail plasmids (RTEP or KTAP). At 24 h after transfection, cell lysates were harvested and used to perform GFP pull-down assays using GFP-trap beads, followed by western blot using anti-14-3-3ε antibodies. **C** 293T cells were transfected with wild-type Tail-GFP plasmids (WT) and EGFP-tagged Tail-GFP mutant plasmids (RTEP or KTAP) for the indicated time points. Whole-cell lysates were collected and probed with anti-GFP, anti-p62, anti-LC3, and anti-β-actin (loading control) antibodies for western blot analyses.

14-3-3ε is a multifunctional protein involved in innate immunity, protein trafficking, cancer development, aggresome formation, and other activities [34]. We previously reported that NSP2 interacts with 14-3-3ε to promote the formation of aggresomes. Thus, the role of 14-3-3ε in NSP2-induced autophagy was investigated. First, the interaction between 14-3-3ε and NSP2 truncations was studied and the results revealed co-localization of 14-3-3ε and the tail domain of NSP2 (Figure 5A). To further confirm an interaction between the two proteins, GFP pull-down assays were performed and the results show that 14-3-3ε was immunoprecipitated by the NSP2 tail region (Figure 5B). On the basis of these results, we focused on the role of 14-3-3ε in aggrephagy caused by the NSP2 tail region.

It has been reported that 14-3-3ε binds NS3 of dengue virus through the RIEP motif [46]. A similar ^991^RTAP^994^ motif is also found in the tail region of NSP2. Thus, two mutant plasmids of KTAP (R991K) and RTEP (A993/E) were constructed. RTEP could bind 14-3-3ε with similar affinity to WT (RTAP), while the binding of KTAP to 14-3-3ε was greatly decreased (Figure 5B). These results reveal that the R in the RTAP motif plays a key role in binding between the NSP2 tail domain and 14-3-3ε. The mutant plasmids were then transfected into 293T cells. Western blots revealed that expression of KTAP decreased the LC3-II/LC3-I ratio and increased the level of p62. Conversely, expression of RTEP increased the LC3-II/LC3-I ratio and decreased the level of p62, similar to the findings for expression of the WT tail. Thus, these results show that 14-3-3ε could promote the occurrence of autophagy.

## Discussion

Substantial evidence has confirmed that PRRSV-2 infection induces autophagy to promote viral replication [41–43, 47], but there is little information on the mechanisms via which this occurs. In this study, we found that NSP2 can induce autophagy which depends on the aggresome formation. NSP2 is a multifunctional protein with a complex structure. The size of NSP2 cleavage products varies between PRRSV-2 strains because of variable cleavage sites at its C-terminus. This variability in cleavage sites explains the differing and sometimes contradictory functions of NSP2. However, strong proof has been established for cleavage sites at or near the G1196/G1197 dipeptide (numbered according to the NSP2 sequence of PRRSV-2 strain VR-2332) [5, 11, 12]. In this study, NSP2 containing 1166 amino acids (1–1166, numbered according to the NSP2 sequence of HP-PRRSV strain TA-12) was used to study its function. Cells have evolved the ability to accommodate different types of protein inclusions by targeting them for clearance via different autophagy pathways [48]. As foreign inclusions, virus particles and viral proteins are often aggregated in cells. The presence of large amounts of viral proteins in cells results in the formation of aggresomes, which are then selectively degraded via autophagy [37]. We found that NSP2 can induce the formation of aggresomes.

To determine whether NSP2-induced aggresomes are degraded via autophagy, we first studied the relationship between NSP2 and autophagy. On the basis of changes in the LC3-II/LC3-I ratio and levels of p62, as well as co-localization of LC3 and NSP2 and the appearance of double-membrane phagophores (Figure 1), we concluded that NSP2 can induce autophagy.

To determine whether NSP2-induced autophagy is dependent on the formation of aggresomes, we used nocodazole to inhibit the formation of aggresomes. The results show that nocodazole can inhibit NSP2-induced autophagy, which implies that the process depends on the formation of aggresomes (Figure 2). Thus, PRRSV-2 can induce aggresome formation (Figure 3) via expression of NSP2.

Addition of nocodazole to PRRSV-2 -infected Marc-145 cells significantly decreased the LC3-II/LC3-I ration and levels of p62. These findings are contradictory to those found during PRRSV-2 -induced autophagy. Multiple viral proteins are produced during PRRSV-2 infection and the decrease in p62 levels could be related to blocking of autophagy due to cleavage of NSP2 protein, together with other autophagy proteins, by caspases or viral proteases [49]. As mentioned above, NSP2 contains four domains. To determine which domain(s) contribute to the induction of aggrephagy, four truncated NSP2 constructs were transfected into cells. Subsequent experiments revealed that the TM and tail domains participate in the formation of aggrephagy (Figure 4). The precise domain(s) of NSP2 that induce autophagy were also investigated. The results also indicate that the TM and tail domains can induce autophagy (Figure 3). Taken together, the results indicate that the TM and tail domains of NSP2 can induce aggrephagy.

The 14-3-3 proteins are a family of conserved regulatory molecules that can bind a multitude of functionally diverse signaling proteins, including kinases, phosphatases, and TM receptors. Of seven isoforms, 14-3-3ε interacts with viral proteins and plays a role in the innate immune response induced by viral infections [38, 50–53]. Our previous study showed that 14-3-3 interacts with NSP2 and that 14-3-3ε acts as a proviral factor during HP-PRRSV infection [39]. Thus, the specific domain(s) of NSP2 that interact with 14-3-3ε were investigated using co-localization assays. The results revealed that the HV and tail domains co-localize with 14-3-3ε (Figure 5A). As the TM and tail domains of NSP2 can induce aggrephagy, interaction of the tail domain with 14-3-3ε was investigated (Figure 5B). The ^991^RTAP^994^ motif, which is similar to the NS3 domain of dengue virus, was identified in the tail domain of NSP2 [46]. To confirm the specific amino acids that contribute in the interaction between 14-3-3ε and the tail domain, R991K and A993/E mutations were introduced into the RTAP motif. These experiments revealed that the R991K mutant decreased the interaction and autophagy. The results also indicate that 14-3-3ε can bind the ^991^RTAP^994^ motif to promote autophagy. Thus, PRRSV-2 induces autophagy to facilitate infection, which may imply that 14-3-3ε functions as a proviral factor.

In vitro, primary pig macrophages (e.g. alveolar macrophages), African green monkey kidney derived cells, such as Marc-145 or modified cell lines are susceptible for the production of PRRSV [54, 55]. In this study, 293T, BHK21, Vero E6 cells were employed, while no results were showed on the alveolar macrophages or other types of swine-originated cell lines because of their hardness to be transfected.

In conclusion, NSP2 can induce aggrephagy and the C-terminal domain contributes to this process. The tail domain of NSP2 plays a key role in inducing aggrephagy by binding 14-3-3ε. These findings provide information on the function of the C-terminal domain of NSP2.
